# Effects of programmatic interventions to improve the management of latent tuberculosis: a follow up study up to five months after implementation

**DOI:** 10.1186/s12889-021-10195-z

**Published:** 2021-01-21

**Authors:** Mercedes Yanes-Lane, Anete Trajman, Mayara Lisboa Bastos, Olivia Oxlade, Chantal Valiquette, Nathalia Rufino, Federica Fregonese, Dick Menzies

**Affiliations:** 1 Research Institute McGill University Health Centre, Montreal, Quebec, Canada; 2 Internal Medicine Graduate Program, Federal University of Rio de Janeiro, Rio de Janeiro, Brazil; 3 Social Medicine Institute, State University of Rio de Janeiro, Rio de Janeiro, Brazil; 4 McGill International TB Centre, McGill University, Montreal, Quebec, Canada; 5 Vector Bourne Diseases Department, Oswaldo Cruz Institute, Rio de Janeiro, Brazil

**Keywords:** Latent tuberculosis, Program evaluation, Cascade of care, Tuberculosis preventive therapy, Cluster randomized trial, Health care workers, Household contacts

## Abstract

**Background:**

Less than 19% of those needing tuberculosis (TB) preventive treatment complete it, due to losses in several steps of the cascade of care for latent TB infection. A cluster randomized trial of a programmatic public health intervention to improve management of latent TB infection in household contacts was conducted in Rio de Janeiro. Interventions included contact registry, initial and in-service training, and a TB booklet. We conducted a follow-up study starting one month after the conclusion of this trial, to measure the effect of interventions implemented, and to identify remaining barriers and facilitators to latent TB infection treatment, from different perspectives.

**Methods:**

In two health clinics in Rio de Janeiro that received the interventions in the trial, data for the latent TB infection cascade of care for household contacts was collected over a five-month period. The number of household contacts initiating treatment per 100 index-TB patients was compared with the cascade of care data obtained before and during the intervention trial. Semi-structured open-ended questionnaires were administered to healthcare workers, household contacts and index-TB patients regarding knowledge and perceptions about TB and study interventions.

**Results:**

In this follow-up study, 184 household contacts per 100 index-TB patients were identified. When compared to the intervention period, there were 65 fewer household contacts per 100 index-TB patients, (95% CI -115, − 15) but the number starting latent TB infection treatment was sustained (difference -2, 95% CI -8,5). A total of 31 index-TB patients, 22 household contacts and 19 health care workers were interviewed. Among index-TB patients, 61% said all their household contacts had been tested for latent TB infection. All health care workers said it was very important to test household contacts, and 95% mentioned that possessing correct knowledge on the benefits of latent TB infection treatment was the main facilitator to enable them to recommend this treatment.

**Conclusion:**

In this follow-up study, we observed a sustained effect of interventions to strengthen the latent TB infection cascade of care on increasing the number of household contacts starting latent TB infection treatment.

**Supplementary Information:**

The online version contains supplementary material available at 10.1186/s12889-021-10195-z.

## Background

According to the World Health Organization (WHO), tuberculosis (TB) is still one of the top 10 causes of death worldwide. Although Brazil has an estimated TB incidence rate of 45 per 100,000 people, making it one of the 30 countries with the highest TB burden, the incidence rate in Rio de Janeiro is more than double the national estimate (93.7 per 100,000 thousand) [1, 2].

In order to reach the targets of the End TB strategy of reducing deaths by 95% and cases by 90% by 2035, [3] TB programs need to make significant efforts to provide continuous essential TB services, including preventive and curative treatment for TB. Tackling the reservoir of latent TB infection (LTBI) has been estimated, from modelling studies, to be the most effective way of reducing TB incidence [4]. As such, one of the pillars of the End TB strategy is integrated patient-centred TB care and prevention, which includes identifying and treating high-risk individuals with LTBI in order to reduce progression to active disease. Household contacts (HHC) of TB patients are included among this group of high-risk individuals [5]. During the 2018 United Nations High Level Meeting on TB, world leaders agreed on a resolution to identify and provide LTBI treatment to four million children under  five years of age and 20 million other HHC by 2022 [6]. However, currently, less than 19% of those needing LTBI treatment complete it, with many losses throughout the cascade of care, including at the stages of identification, diagnosis and treatment [7].

To better inform setting-specific strategies, research from the perspective of health care providers and patients is needed. In a preceding trial (ACT4), [8] interventions were implemented to strengthen key steps in the LTBI cascade of care with the goal of increasing the number of HHC initiating LTBI treatment in Brazil [9]. The primary objective of this follow-up study was to compare the number of HHC identified and initiating LTBI treatment after the trial interventions had ceased to the number identified and initiating LTBI treatment before the trial and during the intervention phase of the trial.

Additional objectives were to identify current barriers and facilitators for linkage to care, and for accepting and initiating LTBI treatment from the perspectives of HHC, health care workers (HCW) and index-TB patients, as well as identifying the acceptability of trial interventions from a HCW perspective.

## Methods

### Study setting

Brazilian guidelines for TB control and prevention recommend isoniazid 300 mg/day for 6 or 9 months in HHCs of all ages who have a positive tuberculin skin test (TST) or positive Interferon-Gamma Release Assay (IGRA). Rifampicin may be used in cases were isoniazid is not recommended, and is preferred for children under 10 years of age, adults over 50 years of age, and those with liver diseases. All treatments for LTBI are self-administered and LTBI is not a compulsory notifiable condition [10].

This study was carried out in two clinics where the intervention trial had previously been implemented. Both clinics are run by the Department of Health of Rio de Janeiro and were set in urban areas [9].

### Study periods

The intervention trial was conducted from May to October of 2018. The follow-up study consisted of two components. Firstly, we conducted a retrospective analysis of the cascade of LTBI care of HHC of index-TB patients diagnosed between November 1, 2018 to March 31, 2019. Secondly, questionnaires were administered to TB patients, HHCs and HCWs between May 7, 2019 to July 4, 2019.

### Intervention trial

The intervention trial consisted of a rapid public health evaluation to identify barriers to LTBI treatment for HHC of index-TB patients, followed by site specific selection of strengthening activities and implementation of interventions to the barriers [9].

In Rio de Janeiro, Brazil, LTBI program strengthening interventions consisted of: (i) initial training of HCWs by a TB physician covering all steps of the LTBI cascade of care, (ii) intensified in-service training of HCWs provided by an infectious disease physician (weekly visits for the first 2 months, then every two weeks for  two months, then once a month), (iii) development and use of a contact registry to facilitate a cascade analysis to support the in-service training of HCWs, (iv) leaflets with educational information for index-TB patients and their contacts and (v) educational material developed for health care workers (TB booklet) [9].

After October 2018, all trial interventions were stopped. This meant cessation of in-service training, provision of leaflets to HHC and index patients, and no further on-site visits by research staff.

### Follow-up study

For the cascade of LTBI care, information on HHCs was obtained from the TB registry at each clinic. After diagnosis of index-TB patients, information on the number of HHCs recorded at each of the following steps was abstracted: HHC identification, initial assessment (TST application and measurement), medical evaluation (consultation with a doctor or nurse, chest radiograph, sputum testing), and HHC initiating treatment (Supplementary Fig. 1). Only HHC of new microbiologically confirmed pulmonary index-TB patients (using either AFB, TB culture, Xpert MTB/RIF or a combination thereof) diagnosed between November 1, 2018 and March 31, 2019 were included in this cascade analysis. Information on the LTBI regimen participants received was not recorded.

In the follow-up study, interviewer-administered open-ended structured questions were applied to TB patients, HHCs and HCWs. TB patients were eligible to be interviewed if they had confirmed (as defined above), or clinical pulmonary TB (defined as a TB diagnosis based on chest X-ray abnormalities and suggestive signs and symptoms, with negative or absent microbiological test results). A HHC was defined as someone who slept in the same house at least one night per week, or spent more than one hour in the house at least five days per week, on average, with an index-TB patient, over the preceding  three months, and in whom active TB has been ruled out. This includes child HHCs, in which case the parents or legal guardians were interviewed. HCWs were defined as doctor, nurse, auxiliary nurse or community health agent that assisted TB patients in either of the two clinics. All HCWs were employed by the Municipal Department of Health of Rio de Janeiro and were not funded or received any incentive from the research funds.

For the questionnaires on barriers and facilitators, a consecutive sample of all TB patients and their HHCs presenting to both clinics from May 7, 2019 to July 4, 2019 were invited to participate. The health clinic directors identified HCW from the TB programs; all were approached and accepted to participate.

For the questionnaires, semi-structured knowledge, attitudes and practices questionnaires were adapted from those used in the ACT4 trial (Supplementary Methods 1, 2, 3). For HHCs and TB patients, questions focused on perspectives and perceptions about the identification of contacts and reasons why linkage to LTBI care was or was not achieved. For HCWs, open-ended questions related to motivation for contact tracing and continuation of activities implemented in the intervention trial were used. All questionnaires were interviewer-administered in the participating health clinics. All interviewees were 18 years of age or older. Written informed consent was provided by all participants prior to data gathering. For participants who could not read or write, the informed consent form was read to them and verbal consent as well as their fingerprint in place of signing was provided. If a family member was accompanying the interviewee, then they were asked to read the informed consent to the patient prior to agreeing to participate.

### Outcomes

The two primary outcomes were the number of HHCs identified and the number of HHCs initiating LTBI treatment within  three months of diagnosis of the index-TB patient. Both outcomes from the follow-up study (November 2018 to March 2019) were compared to: (i) the pre-trial intervention period (July–December 2017), and (ii) the intervention period of the trial (May–October 2018). Outcomes were presented per 100 index-TB patients.

Secondary outcomes included: current barriers and facilitators to LTBI linkage to care, and acceptance and initiation of treatment identified by index-TB patients, HHCs and HCWs; and identifying the acceptability of study interventions from a HCW perspective.

### Analysis

A quasi-Poisson regression model (accounting for over dispersion), with identity link, was used to compare the cascade of care data (number of HHC identified and number of HHC starting LTBI treatment) between the three time periods.

For questionnaire responses, open-ended questions were transcribed from audiotapes and coded by two independent reviewers (MYL, MLB) into common themes. Disagreements were resolved by consensus. The results from these common themes are presented as frequencies and proportions. We compared the responses of HHCs and TB patients from the intervention trial questionnaires to the follow up study questionnaires using the Mantel-Haenszel method for adjusted odds ratio and Wald method for the confidence intervals.

Data analysis was performed using the statistical package R version 3.5.1 (R Foundation for Statistical Computing, Vienna, Austria) and Microsoft Office Excel 2016 (Microsoft Corporation; Redmond, WA).

### Ethical approval

The study was approved in Rio de Janeiro by the Municipal Health Ministry (CAAE 38278214.3.1001.5279) and by the McGill University Health Centre ethical review board (15–291-MUHC).

#### Role of funding source

This study was funded by the Canadian Institute of Health Research, grant number FDN-143350. The funder had no role in study design, data collection, data analysis, data interpretation, or writing of the report. The corresponding author had full access to all the data in the study and had final responsibility for the decision to submit for publication.

## Results

### Cascade of care analysis

The number of index-TB patients and their HHCs, identified during the pre-trial, intervention and follow-up periods were 41 and 78, 57 and 142, and 38 and 70 respectively. This translates to 190, 249 and 184 HHCs identified per 100 index-TB patients in each period. The number of HHCs identified in the follow-up study was 65 fewer (95%CI − 114 to − 15) per 100 index-TB patients compared to during the intervention trial period. From the 70 HHCs identified in the follow-up study (184 HHCs per 100 index-TB patients), 18 (translating to 47 per 100 index-TB patients) initiated LTBI treatment. When comparing the number of HHCs initiating LTBI treatment in the follow-up study to to the intervention period, there was a similar number of HHCs initiating treatment (difference − 2, 95%CI − 8 to 5) per 100 index-TB patients (Table 1).

**Table 1:** Effectiveness of the intervention on different outcomes in the cascade of care

Measurements	Pre-trial intervention period (July–December 2017)	Intervention period of the trial (March–October 2018)	Follow-up study (November–March 2019)
**Number of index-TB patients**	41	57	38
***Total number of HHCs identified (Number per 100 index-TB patients)***^a^	78 (190)	142 (249)	70 (184)
Difference in number per 100 index-TB patients between follow-up study and earlier periods (95% CI)	−6 (− 47 to 34)	−65 (− 115 to − 15)	–
***Total number of HHCs initiating LTBI treatment (Number per 100 index-TB patients)***^a^	2 (5)	28 (49)	18 (47)
Difference in number per 100 index-TB patients between follow-up study and earlier periods (95% CI)	42 (22 to 63)	−2 (− 8 to 5)	–

Notes: 95%CI, 95% confidence interval; ^a^weighted by number of TB patients

**Fig. 1:**
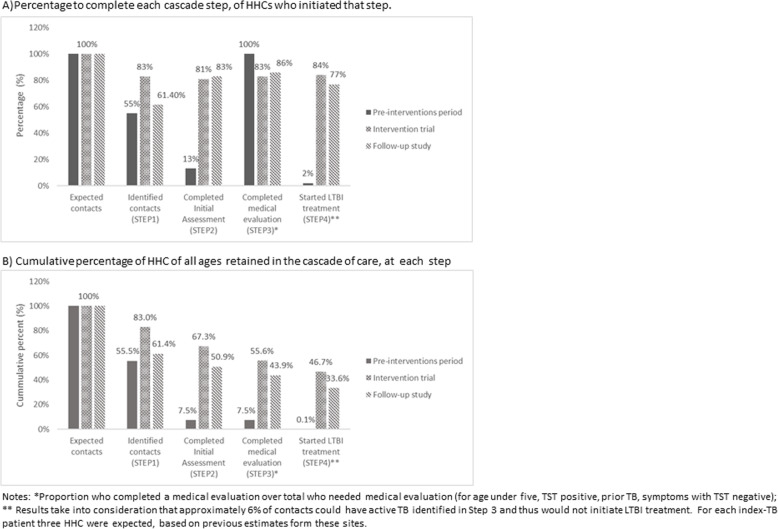
Percentage of HHC of all ages at each step of the cascade of care, during the pre-interventions period, intervention trial, and follow-up study

When looking at the percentage of HHC completing each step of the cascade of those who entered that step (Fig. 1), HHC identification during the follow-up period dropped to similar levels as the pre-interventions period. However, completion of each step of the cascade of care after HHC identification was similar to during the intervention period, including LTBI treatment initiation.

### Questionnaires

A total of 31 TB patients, 22 HHCs and 19 HCW were interviewed during the follow-up study. The mean age (SD) of TB patients was 43 (15.6) years, and of HHCs 46 (16.1) years. No HHCs under the age of 18 were found. The majority of HCWs were female (89%), whereas only 26 and 45% of TB patients and HHCs were female.

The main barriers and facilitators identified by TB patients, HHCs and HCWs are summarized in Table 2.

**Table 2:** Barriers and facilitators identified in TB patient questionnaires, HHC questionnaires and HCW questionnaires

Step in the cascade of LTBI care	Barriers n (%)	Facilitators n (%)
**Barriers and facilitators identified in TB patient questionnaires**		
HHC identification	Not encouraging HHC to be tested 3 (10%)	Counselling from health professionals on LTBI testing in HHC 30 (97%)
	Disbelief in LTBI 2 (6%)	Concern about HHC falling ill with TB 29 (93%)
		Correct knowledge of TB transmission 24 (77%)
Completion of initial assessment and medical evaluation of HHC	Too busy, inconvenient location or working times of the clinics 4 (13%)	Knowledge of TB (as a precaution/fear) 13 (42%)
	Disbelief in LTBI 2 (6%)	Advice by a health professional 6 (19%)
LTBI treatment initiation	Disbelief in LTBI/ no symptoms/ not a priority 7 (23%)	Advice by a health professional 2 (6%)
**Barriers and facilitators identified in HHC questionnaires**		
HHC identification		Correct knowledge of TB transmission 18 (82%)
		Concern about falling ill with TB 18 (82%)
Completion of initial assessment and medical evaluation of HHC	Too busy/not a priority 8 (37%)	Advice by a health professional 12 (55%)
LTBI treatment initiation	Lack of trust in health professionals 1 (5%)	Concern about falling ill with TB 7 (32%)
		Knowledge of TB (prevention/ protection) 21 (95%)
**Barriers and facilitators identified in HCW questionnaires**		
HHC identification		Correct knowledge of LTBI identification in children < 5 years 9 (50%)
		Correct knowledge of the importance of LTBI testing 19 (100%)
Completion of initial assessment and medical evaluation of HHC	Not having access to TST, chest x-ray and INH 1 (5.5%)	
LTBI treatment initiation		Correct knowledge of the benefits of LTBI treatment 18 (95%)

Notes: Blank cells indicate that no barriers were identified

When asked if all their HHCs had been tested, 39% of TB patients said no, the main reasons being that HHCs were too busy to get tested, that they will get tested later and that their HHCs do not believe in the disease or do not feel sick. Among TB patients, 87% said that all or most of their questions regarding TB had been answered by a health professional, and 90% said that they had encouraged their HHCs to be tested. When HHC were asked if they had been checked for LTBI or if they were planning on getting checked, 37% said no, the main reason being that they were too busy, could not get time off work or that they would do it later. When asked if they would take LTBI treatment if recommended, only one HHC said that they would not, because they do not like going to the doctor or taking medication. The main facilitator identified by HCWs, was attitude of HCWs towards LTBI. All (100%) of HCWs said it was very important to test HHCs for LTBI. Almost all (95%) mentioned that possessing correct knowledge on the benefits of LTBI treatment was the main facilitator to enable them to recommend this treatment.

When comparing the answers from the TB patient and HHC questionnaires from the follow up study to the intervention trial, we observed that a non-significantly higher proportion of HHCs were willing to be tested (63.6% (14/22) vs 52.6% (10/19)) and to take treatment for LTBI if necessary (95.5% (21/22) vs 85% (17/20). A non-significantly higher proportion of TB patients was also informed about HHC testing for LTBI (96.8% (30/31) vs 87.5% (21/24). (Supplementary Table 1).

With regards to the acceptability of the interventions, the majority of HCWs (61%) considered the TB booklet to be an accessible tool for solving day-to-day queries on LTBI diagnosis and treatment. Initial (56%) and in-service training (39%) were considered important for changing the attitude and beliefs about LTBI. The main themes identified in the open ended questions from HCW questionnaires can be found in Supplementary Table 2. Almost all (95%) of HCWs said it was useful to have a member of the research team helping in LTBI management (training and in-service training), while 26% of HCWs mentioned that it would be useful to have personnel exclusively focused on LTBI care. Sixty-eight percent said that they felt more prepared to treat TB and LTBI patients after interventions had been implemented.

## Discussion

This study evaluating the long term effects of programmatic public health interventions showed a sustained improvement in the number of HHCs initiating LTBI treatment, up to  five months after the interventions had been stopped by research staff. The main facilitator to linkage to care, acceptance and initiation of treatment identified by HCWs, TB patients and HHCs was knowledge of LTBI diagnosis and treatment.

In this follow-up study, we identified losses of HHCs at the identification step of the cascade of care. However, this did not affect the number of HHCs initiating LTBI treatment per 100 index TB-patients, which remained similar to the intervention trial. Previous studies of the cascade of LTBI care in Brazil had also demonstrated the highest losses in the initial steps (identification and investigation) [11, 12]. As mentioned by HCWs in the questionnaires, interventions such as training, TB booklet and in-service training helped improve contact identification and treatment initiation. Notwithstanding, HCWs also mentioned that there is a need for personnel exclusively dedicated to LTBI care and that having someone from the research team providing them with feedback of their performance was highly useful. Information from this study provides insights as to which interventions are acceptable to HCW and can be adopted as routine practice by the National TB Program (NTP), as well as signaling areas which could be improved upon. Although study led activities have ended, the public health interventions which were implemented, such as educational material, formal and in-service training, were designed to be replicable and sustainable by TB clinics.

The quantitative assessment of the cascade of care, which shows ongoing losses at the identification step – despite its improvement from the pre-interventions period, contrasted with the qualitative assessment from the interviews, in which very few barriers towards HHC identification were found among all the interviewed groups. This may be due to the effectiveness of the interventions in overcoming previous barriers, although social desirability and selection biases cannot be ruled out, as all questionnaires were interviewer-administered and only patients who attended the clinics were interviewed.

From the TB patient questionnaires, we see that most of the patients interviewed encouraged their HHCs to get tested and that most of them were worried that someone else in their household would get sick with TB. Both the findings from HHCs and TB patients indicate that a strong support network, including family and HCWs, is an important enabler for linkage to LTBI care, and acceptance and initiation of treatment, as has been shown in other studies [13–15].

Given that a shift in HCW knowledge, attitude and practices plays a vital role in decreasing the losses at different stages of the cascade of care, [16, 17] it may be warranted to include interventions focused on continuous training tailored to site-specific context, [18] within all TB prevention programs.

This study is not without its limitations. It was not possible to interview the same participants from the intervention study. However, our objective was to measure the effects from the interventions, which is reflected in the cascade of care as well as in responses from new participants. The power to detect statistical differences is limited by our sample size, which in itself is limited by the total number of HCWs, TB patients and HHCs at each clinic. The period of time between end of the intervention trial and measurement of the cascade of LTBI care may not be long enough to capture long term effects of said interventions. Nonetheless, we consider that the time period between end of interventions to the new questionnaires provides a washout period that allows us to evaluate the persistent effects of interventions.

One of the main strengths of this study is that we were able to evaluate the effects of interventions under programmatic conditions, thus providing useful information for  NTPs. Another strength of this follow-up study is that HCWs, HHCs and index-TB patients were included to gain an understanding of the LTBI cascade of care from the perspective of all parties involved [7].

## Conclusion

In summary, the number of HHCs initiating LTBI treatment, adjusted by the number of index-TB patients, in the five months after the termination of a programmatic trial, was similar to the number during the intervention period of the trial, and significantly higher than the pre-interventions period of the same trial. This suggests a sustained effect of the interventions.

## Supplementary Information

**Additional file 1: Supplementary Fig. 1**. Steps in the LTBI cascade of care. **Supplementary Methods 1**. Questionnaire for adult household contacts. **Supplementary Methods 2**. Questionnaire for TB patients. **Supplementary Methods 3**. Questionnaire for health care workers. **Supplementary Table 1**. Odds of answering “yes” to a given question in the interventions trial’s questionnaires compared to the odds of answering “yes” in the follow-up study’s questionnaires. **Supplementary Table 2**. Main themes identified in open ended questions from the HCW questionnaires

## Data Availability

The datasets used and/or analysed during the current study are available from the corresponding author on reasonable request.

## Abbreviations

AFB: Acid fast bacilli
HCW: Health care worker
HHC: House hold contact
IGRA: Interferon-gamma release assay
LTBI: Latent TB infection
NTP: National TB program
TB: Tuberculosis
TST: Tuberculin skin test
WHO: World health Organization
