# Supplementary material for: Effects of programmatic interventions to improve the management of latent tuberculosis: a follow up study up to five months after implementation
Source: BMC Public Health. 2021 Jan 21;21:177. doi: 10.1186/s12889-021-10195-z (PMC7819253; doi:10.1186/s12889-021-10195-z)
Supplement: Supplementary file 1 — Additional file 1: Supplementary Fig. 1. Steps in the LTBI cascade of care. Supplementary Methods 1. Questionnaire for adult household contacts. Supplementary Methods 2. Questionnaire for TB patients. Supplementary Methods 3. Questionnaire for health care workers. Supplementary Table 1. Odds of answering “yes” to a given question in the interventions trial’s questionnaires compared to the odds of answering “yes” in the follow-up study’s questionnaires. Supplementary Table 2. Main themes identified in open ended questions from the HCW questionnaires [file 12889_2021_10195_MOESM1_ESM.docx]

**Supplementary material**

**Supplementary Figure 1. Steps in the LTBI cascade of care.**

Notes: HHC, household contact; TST, tuberculin skin test; LTBI, latent TB infection.

**Supplementary Methods 1. Questionnaire for adult household contacts.**

1. Sex:

|___| Male

|___| Female

1. What year were you born in?

|___|___|___|___|

1. Where would you would normally go first if you had a health problem?

|___|Traditional healer/Alternative medicine

|___|Pharmacy

|___|Private doctor/clinic/hospital

|___|Public doctor/clinic/hospital

|___|TB clinic or TB hospital

|___|Other (specify) _______________________

1. Why do you usually go for care at the place you listed?

| Health clinic/ health care worker related | Patient related |
| --- | --- |
| \|___\|Free/ affordable/ low Prices | \|___\|Advice from family, friends, co-workers |
| \|___\|Convenience - location, time hours  of operation, short wait time | \|___\|Intentionally go to a place where I will  be anonymous (stigma) |
| \|___\|Good quality of care in past, well equipped |  |
| \|___\|Trust staff |  |
| \|___\|Previous visits to the clinic |  |
| \|___\|Other (specify):_______________________________________________________ | |

1. How much do you worry about getting sick with TB?

|___|A great deal

|___|Quite a bit

|___|Somewhat

|___|Very little

|___|Not at all

1. How do you think TB is transmitted from one person to another?

|___|Indirect contact (through air) - living in same house or room / breathing

the same air

|___|Indirect contact (through objects) - same dishes / cups / toothbrush

|___|Direct physical contact- touching / hugging / kissing

|___|Fate / bad luck / up to God / no control / will just happen

|___|Not taking care of yourself- Becoming too tired / exhausted / poor diet

|___|Bad habits / unhealthy lifestyle / smoking / drinking alcohol / drugs

|___|Unclean living environment

|___|Travel/ visit to another country / return visit to home country

|___|Other (specify): ____________________________________

1. Were you checked for TB, or will you be checked soon for TB?

| \|___\| Yes, why? | \|___\| No, why? |
| --- | --- |
| \|___\|Worried/concerned about personal  health, fear of TB | \|___\|Too busy, could not take time off, would take too much time |
| \|___\|For prevention/ health maintenance | \|___\|Not convenient - location, time, hours of operation, long wait times |
| \|___\|Felt sick, had symptoms, needed to be  checked/ tested | \|___\|Cost - for travel, time off work  \|___\|Cost - for testing, visit to clinic |
| \|___\|Advice of health care worker | \|___\|Do not need, do not believe, not a priority/ I am not sick / I have no symptoms of TB |
| \|___\|Advice of friends or family or co- workers  (**to go**) | \|___\|Was not told, not informed, did not  understand, did not know where to go |
| \|___\|Out of respect for the active TB case | \|___\|Advice of family or friends or co-workers (to **not** go) |
| \|___\|Home visit was made | \|___\|No-one to go with me / cannot go alone / need support |
| \|___\|Other (specify): ______________________  ________________________________________ | \|___\|Afraid of stigma, discrimination |
|  | \|___\|Other (specify): ________________________  __________________________________________ |

1. Did you go (or are you planning to go) to the clinic/hospital for a medical evaluation for TB?

| \|___\|Yes, why? | \|___\|No, why? |
| --- | --- |
| \|___\|Worried/concerned about personal  health, fear of TB | \|___\|Too busy, could not take time off, would take too much time |
| \|___\|For prevention/ health maintenance | \|___\|Not convenient - location, time, hours of  operation, long wait times |
| \|___\|Felt sick, had symptoms, needed to  be checked / tested | \|___\|Cost - for travel, time off work  \|___\|Cost - for testing, physician visit |
| \|___\|Advice of health care worker | \|___\|Do not need, do not believe, not a priority/ I am not sick / I have no symptoms of TB |
| \|___\|Advice of friends or family or co- workers (**to go**) | \|___\|Do not like the clinic, staff are not friendly,staff not knowledgeable, staff not helpful |
| \|___\|Out of respect for the active TB case | \|___\|Was not told, not informed, did not  understand, did not know where to go |
| \|___\|Other (specify): ______________________ ________________________________________ | \|___\|Advice of family or friends or co-workers (to **not** go) |
|  | \|___\|No-one to go with me / cannot go alone / need support |
|  | \|___\|Afraid of discrimination/ stigma |
|  | \|___\|Other (specify): __________________________  ____________________________________________ |

9.Have you seen the flyers of Latent TB infection?

Yes □

No □

Please give the reasons for your answer. ­­­­­­­­­­­­­­

1. If the doctor/nurse advised you to take medication to treat a TB infection, did you agree to take it? (or, if they will advise you to take medications, would you agree to take it?).

| \|___\|Yes, why? | \|___\|No/Undecided, why ? |
| --- | --- |
| \|___\|Worried/concerned about personal health, fear of TB | \|___\|Too busy, could not take time off, would take too much time |
| \|___\|For prevention/ health maintenance | \|___\|Not convenient - location, time, hours of operation, long wait times |
| \|___\|Believe/trust doctor, nurse, or other worker | \|___\|Cost - for travel, testing, physician visit, time off work, medications |
| \|___\| Protect others (family / friends /co-workers) from getting TB | \|___\|Do not need, do not believe, not a priority/I am not sick, I have no symptoms of TB |
| \|___\|Protect myself, protect my own health, stay healthy, prevent illness | \|___\|Fear of side effects of treatment, dislike antibiotics/pills, fear blood tests |
| \|___\|Other (specify): ______________________  ________________________________________ | \|___\|Was not told, not informed, did not understand, did not know where to go |
|  | \|___\| Advice of family or friends or co-workers (to not go) |
|  | \|___\|No-one to go with me / cannot go alone / need support |
|  | \|___\|Afraid of stigma, discrimination |
|  |  |

**Supplementary Methods 2. Questionnaire for TB patients.**

1. Sex:

|___| Male

|___| Female

1. What year were you born in?

|___||___||___| |___|

1. Where would you normally go first if you had a health problem?

| Traditional healer/Alternative medicine | \|___\| |
| --- | --- |
| Pharmacy | \|___\| |
| Private doctor/clinic/hospital | \|___\| |
| Public doctor/clinic/hospital | \|___\| |
| TB clinic or TB hospital | \|___\| |
| Other (specify) _______________________ | \|___\| |

1. Why do you usually go for care at the place you listed?

| Health clinic/ health care worker related | Patient related |
| --- | --- |
| \|___\|Free/ affordable/ low prices | \|___\|Advice from family, friends, co-workers |
| \|___\|Convenience - location, time hours of  operation, short wait time | \|___\|Intentionally go to place where I will be anonymous (stigma) |
| \|___\|Good quality of care in past, well equipped | \|___\|Other (specify):___________________ |
| \|___\|Trust staff |  |
| \|___\|Previous visits to the clinic |  |
| \|___\|Other (specify): _________________________  ___________________________________________ |  |

1. How well did the staff of this clinic/hospital answer your questions about your TB diagnosis and treatment?

|___| All of my questions were answered

|___| Most of my questions were answered

|___| Some of my questions were answered

|___| Almost none of my questions were answered

|___| I was not able to ask questions

|___| I did not have any questions

|___| I was shy, embarrassed, not comfortable to ask questions

|___| Other (specify):________________________________________

1. Did someone at the clinic explain to you that your household contacts should be checked or tested for TB?

|___| Yes

|___| No

1. How do you think TB is transmitted from one person to another?

|___|Indirect contact (through air) - living in same house or room / breathing the same air

|___|Indirect contact (through objects) - same dishes / cups / toothbrush

|___|Direct physical contact- touching / hugging / kissing

|___|Fate / bad luck / up to God / no control / will just happen

|___|Not taking care of yourself- Becoming too tired / exhausted / poor diet

|___|Bad habits / unhealthy lifestyle / smoking / drinking alcohol / drugs

|___|Unclean living environment

|___|Travel/ visit to another country / return visit to home country

|___|Other (specify): ____________________________________

1. How worried are you that someone else in your household might get sick with TB?

|___| A great deal

|___| Quite a bit

|___| Somewhat

|___| Very little

|___| Not at all

1. Did you encourage your household contacts to be checked or tested for TB?

| \|___\| Yes, why? | \|___\|No, why? |
| --- | --- |
| \|___\|The contacts were sick, had symptoms of  possible TB (such as cough, fever, night sweats) | \|___\|Disbelief, not sick, not infected, no need for tests or treatment, no proof of TB |
| \|___\|Fear, worried, concerned about TB, concerned about sickness, they might die. | \|___\|Fear of side effects of treatment, dislike antibiotics/pills, fear blood tests |
| \|___\|Advised and believe doctor or nurse or other  worker. Trust doctor/nurse. | \|___\|Shame of TB, stigma of TB, lose face, embarrassment, social consequences |
| \|___\|Protect others. Protect family / friends / co- workers. Prevent transmission | \|___\|Inconvenience - clinic location, clinic hours |
| \|___\|Protect their health, stay healthy, prevent  illness. | \|___\|Cost for travel, time off work, cost for tests / medications, cost for doctor visit. |
| \|___\| Other (specify): ______________________  ________________________________________ | \|___\|The doctor said I am not contagious, no need to have them checked |
|  | \|___\|I did not know the clinic offered testing for household contacts |

Other:

1. Did all of your household contacts get checked/tested for TB?

| \|___\| Yes | \|___\|No, why? |
| --- | --- |
|  | \|___\|Too busy, could not take time off, would take too much time |
|  | \|___\|Not convenient - location, time, hours of  operation, long wait times |
|  | \|___\|Cost - for travel, testing, physician visit, time off work |
|  | \|___\|Do not need, do not believe, not a priority/are not sick, no symptoms of TB |
|  | \|___\|Do not like the clinic, staff are not friendly, staff not knowledgeable, staff not helpful |
|  | \|___\|Was not told, not informed, did not understand, did not know where to go |
|  | \|___\|Not part of family (only a friend/roommate)/not responsible for them |
|  | \|___\|No-one to go with them / cannot go alone / need support |
|  | \|___\|Could not contact/no longer living with us |
|  | \|___\|Afraid of stigma, discrimination |
|  | \|___\|They were weak, old |
|  | \|___\|Other (specify):____________________ |

1. Did any of your household contacts diagnosed with LTBI start preventative treatment for TB?

| \|___\| Yes | \|___\|No, why? |
| --- | --- |
| \|___\| Advised and believe doctor/nurse/other worker | \|___\|Too busy, could not take time off, would take too much time |
|  | \|___\|Not convenient - location, time, hours of  operation, long wait times |
| \|___\| To protect themselves from TB, stay healthy, avoid getting TB/reduce the risk of TB | \|___\|Cost - for travel, testing, physician visit, time off work |
|  | \|___\|Do not need, do not believe, not a priority/are not sick, no symptoms of TB |
| \|___\| To protect others: prevent TB transmission | \|___\|Do not like the clinic, staff are not friendly, staff not knowledgeable, staff not helpful |
|  | \|___\|Was not told, not informed, did not understand, did not know where to go |
| \|___\| Fear, worried, concerned about TB, concerned about sickness | \|___\|Not part of family (only a friend/roommate)/not responsible for them |
|  | \|___\|No-one to go with them / cannot go alone / need support |
| \|___\| Treatment is effective and safe | \|___\|Could not contact/no longer living with us |
|  | \|___\|Afraid of stigma, discrimination |
| \|___\|Other (specify):____________________ | \|___\|They were weak, old |
|  | \|___\|Other (specify):____________________ |

1. Since your TB started, have you or your family had any financial problems?

|___| No financial problems

|___| Sold some of your belongings

|___| Removed a child from school

|___| Took out a loan/ borrowed money (from bank or family/friends)

|___| Could not pay rent, or had to change housing as could not afford current housing

|___| Lost your job

|___| Could not afford to buy food for yourself/your family

|___| Other (specify): ­­­­­­­­­______________________________________

**Supplementary Methods 3. Questionnaire for health care workers.**

1. What is your position at this health facility?

|___| Doctor (*Please specify type if specialist*)*:* _____________________________

|___| Nurse *(Please specify type if specialist*)**:** _____________________________

|___| Auxiliary nurse

|___| Community health agent

|___| Other kind of specialised health care worker (ie. Social worker, etc..):_________

|___| Educator

|___| Other (specify):__________________________________________________

1. How do you think TB is transmitted from one person to another?

|___| Indirect contact (through air) - living in same house or room / breathing the same air

|___| Indirect contact (through objects) - same dishes / cups / toothbrush

|___| Direct physical contact- touching / hugging / kissing

|___| Fate / bad luck / up to God / no control / will just happen

|___| Not taking care of yourself- Becoming too tired / exhausted / poor diet

|___| Bad habits / unhealthy lifestyle / smoking / drinking alcohol / drugs

|___| Unclean living environment

|___| Travel/ visit to another country / return visit to home country

|___| Other (specify): ____________________________________

1. For an adult household contact who has latent TB (positive TST or IGRA), what is the most important thing to do in order to prevent TB disease?

|___|BCG vaccination

|___|Treatment with LTBI medications

|___|Ensure the person with active TB is following instructions to prevent transmission– taking treatment, cough etiquette, wearing a mask

|___|Isolation from the active case: e.g. stay in a different room, different home wearing a mask, not sharing objects for eating, utensils, toothbrush

|___|Healthy lifestyle: rest, sleep, diet, no alcohol, no smoking

|___|Return for re-evaluation if you develop any risk factors (HIV/renal failure/diabetes)

|___|Other (specify): _____________________________________

1. Isoniazid (INH) can be used for 6 or 9 months to prevent active TB. Approximately how effective is a full course of latent treatment (with INH) for preventing active TB?

6 months of treatment with INH |___| %

9 months of treatment with INH |___| %

1. What is the most essential next step once a household contact who is under **5 years age** has been identified?

|___|Refer to another physician or specialist or another centre

|___|Ask about symptoms of active TB (cough, fever, loss weight, short of breath) / and do physical examination for symptoms of active TB

|___|Test for LTBI – with skin test - TST (PPD, Mantoux) / or an IGRA

|___|Chest X-ray

|___| Sputum-gastric aspiration

|___|Would not conduct any tests

|___|Not applicable to their position, not their job, does not do this

|___|Other (specify): __________________________

1. How important do you think it is for contacts of patients with TB to get tested for LTBI?

|___| Very important

|___| Somewhat important

|___| Not important

|___| Very unimportant

1. If you had a patient who was a household contact of an active TB case who had a positive TB skin test (TST) or blood test (IGRA), and active TB was excluded, would you advise them to take LTBI treatment?

| \|___\|Yes , why? | \|___\|No, why? |
| --- | --- |
| ___________________________________________  ___________________________________________  ___________________________________________ | \|___\|INH (or other drugs) not always available |
|  | \|___\|Risk of side effects |
|  | \|___\|Worried about creating drug resistance |
|  | \|___\|I do not have the expertise |
|  | \|___\|It is not an effective treatment |
|  | \|___\|Too much demand already with active TB  patients |
|  | \|___\|Patients not interested/too concerned  about side effects etc. |
|  | \|___\| Other (specify):______________________  ________________________________________ |

1. If you were a household contact of an active TB case, and you had a positive TB skin test (TST) or blood test (IGRA), would you take treatment for latent TB?

Yes □

No □

Please give the reasons for your answer:

1. In your day to day work with patients with LTBI do you have any problems of access to the following:

|___| Chest x-ray

|___| TST

|___| Isoniazid

1. From your perspective as a health care worker, what intervention was **most helpful** for you in your work taking care of patients with latent tuberculosis?

|___| Education (Lectures)

|___| Graphs

|___| In service training

|___| Booklets

|___| Flyers

|___| Registry book (yellow book)

|___| Other­­­­­­­­­­­­­_________________________________________________

1. What do you think about the training that was given, the small booklet and the flyers?

Training Small booklet Flyers

|___| Very useful |___| Very useful |___| Very useful

|___| Somewhat useful |___| Somewhat useful |___| Somewhat useful

|___| Not especially useful |___| Not especially useful |___| Not especially useful

|___| Not useful |___| Not useful |___| Not useful

Please give the reasons for your answer:

1. Do you currently use the contact registry book?

Yes □

No □

Please give the reasons for your answer: ­­­­­­­­­­­­­­

1. Currently the NTP has an indicator for LTBI which is proportion of household contacts who are examined. Do you think this is an appropriate indicator?

Yes □

No □

Please give the reasons for your answer: ­­­­­­­­­­

1. Can you suggest another indicator that might be more useful and appropriate?

Yes □

No □

If yes, which one? ­­­­­­­­­­­­­­­­­­­­­­­

1. Would you think an indicator of the proportion of HHC who start treatment for LTBI would be useful?

Yes □

No □

Please give the reasons for your answer

**Open ended section.**

1. Do you think it was helpful to have someone of the study helping in the daily activities for LTBI treatment?
2. Do You feel that your work with household contacts with LTBI changed before the study and after? How? And why
3. Do you think that the program at this clinic for investigation and treatment of HHC is now effective to prevent TB?
4. What else do you think could be done to improve LTBI identification and treatment?
5. If you were transferred to another facility would you continue to identify, investigate and treat contacts?
6. Did the study change how you think about TB contacts and their management?

**Supplementary Table 1. Odds of answering “yes” to a given question in the interventions trial’s questionnaires compared to the odds of answering “yes” in the follow-up study’s questionnaires.**

| **HHC questionnaires** | | | |
| --- | --- | --- | --- |
| Were you checked for TB, or will you be checked soon for TB | Yes | No | OR (95%CI) |
| Intervention trial | 10 | 9 | 0.63 (0.18-2.22) |
| Follow-up study | 14 | 8 |  |
| If the doctor/nurse advised you to take medication to treat a TB infection, did you agree to take it? (or, if they will advise you to take medications, would you agree to take it?) | Yes | No | OR (95%CI) |
| Intervention trial | 17 | 3 | 0.40 (0.03-4.85) |
| Follow-up study | 21 | 1 |  |
| **TB patient questionnaires** | | | |
| Did someone at the clinic explain to you that your household contacts should be checked or tested for TB? | Yes | No | OR (95%CI) |
| Intervention trial | 21 | 3 | 0.35 (0.03-4.11) |
| Follow-up study | 30 | 1 |  |
| Did you encourage your household contacts to be checked or tested for TB? | Yes | No | OR (95%CI) |
| Intervention trial | 22 | 2 | 0.88 (0.11-6.78) |
| Follow-up study | 28 | 3 |  |
| Did all of your household contacts get checked/tested for TB? | Yes | No | OR (95%CI) |
| Intervention trial | 18 | 6 | 1.89 (0.59-6.12) |
| Follow-up study | 19 | 12 |  |

**Supplementary Table 2. Main themes identified in open ended questions from the HCW questionnaires.**

| **Question** | **Main themes** | **n (%)** |
| --- | --- | --- |
| Do you think it was helpful to have someone from the study helping with LTBI management? (training and in-service training) | Yes | 18 (95) |
|  | Not sure if it was useful/no contact with the study | 1 (5) |
|  | Continuous education | 2 (11) |
|  | Provided new and practical information | 8 (43) |
|  | Helped with day to day management activities | 3 (16) |
|  | Increased awareness | 1 (5) |
|  | Instant answer to questions | 3 (16) |
| What else do you think could be done to improve LTBI identification and treatment? | Have someone only for LTBI | 5 (26) |
|  | Continuous education for HCW | 5 (26) |
|  | TB education campaigns | 4 (21) |
|  | Respirologists | 1 (5) |
|  | Expand clinic hours | 1 (5) |
|  | Someone to follow up on treatment initiation | 1 (5) |
|  | Home visits | 2 (11) |
|  | Nothing to improve | 2 (11) |
| Do you feel that your work with household contacts with LTBI changed before the study and after? How? And why | More prepared to treat TB and LTBI | 13 (68) |
|  | More willing to help co-workers | 1 (5) |
|  | Better patient care | 5 (26) |
|  | Information from training is forgotten over time | 1 (5) |
|  | Greater concern for LTBI | 4 (21) |
|  | Not much changed | 2 (11) |
|  | Decreased number of cases | 1 (5) |
| Do you think that the program at this clinic, for identifying and treating HHC, is now effective in preventing TB? | Yes | 16 (84) |
|  | Not enough to prevent TB | 4 (21) |
| If you were transferred to another facility would you continue to identify, investigate and treat contacts? | Yes | 16 (89) |
|  | Yes if that was the protocol | 2 (11) |
| Did the study change how you think about TB contacts and their management? | Yes, increased contact evaluation, correct protocol with adequate resources | 6 (33) |
|  | Yes | 6 (33) |
|  | Yes but there are times when we don't have the resources (TST, INH) | 1 (6) |
|  | No change in perception only increased knowledge | 2 (22) |
